# Geospatial mapping of timely access to inpatient neonatal care and its relationship to neonatal mortality in Kenya

**DOI:** 10.1371/journal.pgph.0000216

**Published:** 2022-06-30

**Authors:** Paul O. Ouma, Lucas Malla, Benjamin W. Wachira, Hellen Kiarie, Jeremiah Mumo, Robert W. Snow, Mike English, Emelda A. Okiro

**Affiliations:** 1 Population Health Unit, Kenya Medical Research Institute-Wellcome Trust Research Programme, Nairobi, Kenya; 2 Health Services Unit, Kenya Medical Research Institute-Wellcome Trust Research Programme, Nairobi, Kenya; 3 The Aga Khan University, Nairobi, Kenya; 4 Health Sector Monitoring and Evaluation Unit, Ministry of Health, Nairobi, Kenya; 5 Nuffield Department of Clinical Medicine, Centre for Tropical Medicine and Global Health, University of Oxford, Oxford, United Kingdom; University College London, UNITED KINGDOM

## Abstract

Globally, 2.4 million newborns die in the first month of life, with neonatal mortality rates (NMR) per 1,000 livebirths being highest in sub-Saharan Africa. Improving access to inpatient newborn care is necessary for reduction of neonatal deaths in the region. We explore the relationship between distance to inpatient hospital newborn care and neonatal mortality in Kenya. Data on service availability from numerous sources were used to map hospitals that care for newborns with very low birth weight (VLBW). Estimates of livebirths needing VLBW services were mapped from population census data at 100 m spatial resolution using a random forest algorithm and adjustments using a systematic review of livebirths needing these services. A cost distance algorithm that adjusted for proximity to roads, road speeds, land use and protected areas was used to define geographic access to hospitals offering VLBW services. County-level access metrics were then regressed against estimates of NMR to assess the contribution of geographic access to VLBW services on newborn deaths while controlling for wealth, maternal education and health workforce. 228 VLBW hospitals were mapped, with 29,729 births predicted as requiring VLBW services in 2019. Approximately 80.3% of these births were within 2 hours of the nearest VLBW hospital. Geographic access to these hospitals, ranged from less than 30% in Wajir and Turkana to as high as 80% in six counties. Regression analysis showed that a one percent increase in population within 2 hours of a VLBW hospital was associated with a reduction of NMR by 0.24. Despite access in the country being above the 80% threshold, 17/47 counties do not achieve this benchmark. To reduce inequities in NMR in Kenya, policies to improve care must reduce geographic barriers to access and progressively improve facilities’ capacity to provide quality care for VLBW newborns.

## Introduction

In sub-Saharan Africa (SSA), the neonatal period is the most vulnerable time for child survival [1]. Between 1990 and 2015, under-5 mortality declined by 51%, but this rate of decline was slower in the neonatal period, and in 2015, deaths in the first 28 days of life accounted for 37% of all under 5 deaths [2]. Meeting the Sustainable Development Goal 3.2 of 12 or fewer neonatal deaths per 1,000 livebirths [3] in these countries requires accelerated scale-up of effective interventions that target the major causes of newborn deaths. The Lancet series on newborn health estimated that the maximum effect of newborn death reduction can be achieved by ensuring access to adequate interventions delivered during labour and birth, mainly obstetric care (41%) and caring for small and sick newborns (30%) [4].

Access to these interventions is affected by several factors that can be categorised as socioeconomic and cultural [5–7], geographic accessibility [8–10] and receiving adequate treatment once at the facility [11]. Geographic accessibility is significant in SSA because distance is a common challenge in many marginalised communities [12]. Geographic access relates to movement from point of origin to the destination facility with required services and may be affected by various physical barriers. For example, distances may be long, transport may be slow or unavailable, elevation may reduce transport speeds or transport barriers such as forests, lakes and rivers may increase travel distances. These are well-known mobility barriers in SSA and community-based studies have reported their significance in delaying patients from reaching health service providers [13–15].

Existing recommendations suggest ensuring accessibility within 2 hours is achieved for 80% of the population as a key measure of universal health coverage with surgical and obstetric services [16]. Most studies have used straight-line distances as a measure of geographic access, rather than travel times that encompass physical and weather-driven impediments to access [17, 18]. In addition, studies that have assessed geographic access using travel time did not use service availability assessments and this may have overestimated geographic accessibility [19, 20]. However, the recent developments in data collection at facility levels such as service availability and readiness assessments and health information systems provide an opportunity to adjust geographic accessibility metrics for likely facility capacity to provide services based on available resources.

Although the 2-hour metric has been used to measure access to emergency surgical services and obstetrics [19, 21, 22], there have been no studies measuring geographic access to facilities capable of caring for newborns requiring inpatient services. The main causes of neonatal mortality are neonatal sepsis, prematurity and intrapartum related complications [23]. Most of the deaths from these conditions can be prevented with access to high-quality interventions such as intravenous antibiotics, assisted feeding and supplemental oxygen, typically delivered by nurses and overseen by medical doctors. Previous studies show an increase in neonatal mortality as access becomes poor [24–26], but none of these studies has assessed the importance of geographic access to inpatient newborn care on neonatal mortality.

This paper aimed to assess geographic access to hospitals with a minimum set of resources enabling care for VLBW newborns (birthweight < 1,500 g) in Kenya. The country had neonatal mortality rate of 21 per 1,000 livebirths (NMR) in 2018 and one of the countries not on track to achieve SDG 3.3 on reducing NMR to less than 12 by 2030 [27]. VLBW was therefore chosen as a tracer inpatient newborn service because of the high risk of death in this group [28, 29]. Newborns in the VLBW category require careful monitoring by trained health professionals [4], based in inpatient facilities with infant incubators, oxygen, ability to provide caesarean deliveries, space for kangaroo mother care (KMC) and feeding support using nasogastric and intravenous tubes [30, 31]. Here we use reported availability of these time-sensitive interventions to determine which hospitals seem able to provide inpatient obstetric and VLBW care. To improve understanding of geographic access to VLBW hospitals, a cost distance algorithm that accounted for proximity to roads, road conditions, rainfall patterns and transport barriers such as protected areas was used. The aim was to determine the proportion of VLBW newborns living within 2 hours from the nearest VLBW hospital for each of Kenya’s 47 counties. Finally, to evaluate the impact of access to VLBW services on newborn health, the access metrics at county levels were regressed against NMR, while controlling for other confounders. Results from this work are useful in identifying areas where services need to be improved or for targeting alternative interventions for reducing travel time such as maternity waiting homes.

## Materials and methods

### Mapping inpatient newborn hospitals

There is no single definitive census of hospital services in Kenya and therefore a composite of data sources were used to map hospitals able to offer VLBW services. These were the Master Facility List (MFL), Service Availability and Readiness Mapping (SARAM), the Harmonised Health Facility Assessment (HHFA), the Emergency Medicine Kenya Foundation (EMKF) and the District Health Information System (DHIS 2) (Table 1). Thus, the aim was to use these data sources to assemble an inventory of facilities meeting initial criteria for further interrogation of VLBW service availability. The Kenya county shapefiles was obtained from humanitarian data exchange (HDX) website that is maintained by Office for the Coordination of Humanitarian Affairs (OCHA) [32].

**Table 1:** Summary of sources of hospital data, the years they represent, their scope and availability status.

Source	Year	No. of hospitals	Scope	Data availability	Citation
MFL	2018	534	Reports on the health facilities available	Publicly available	[68]
SARAM	2013	512	Service availability for all Facilities in Kenya	Not publicly available	[69]
EMFK	2018	119	Service availability all government hospitals with a functioning theatre	Not publicly available	[36]
HHFA	2018	412	Service availability for sample facilities but included all hospitals	Not publicly available	[70]
DHIS 2	2016 & 2018	289	Reporting rates of number of CS in each hospital	Available on request	[71]

MFL; Master Facility List, SARAM: Service Availability and Readiness Mapping, HHFA: Harmonised Health Facility Assessment, EMKF: Emergency Medicine Kenya Foundation and DHIS 2: District Health Information System

The MFL is a complete, authoritative, up-to-date inventory of health facilities [33]. It is the primary source for facility names, unique code, contact information, location attributes, administrative data facility type, ownership or operational status [34]. The SARAM was undertaken during the implementation of the devolved system of governance to counties in 2013. DHIS2 is the platform for routine health data in the country [35]. The HHFA data was a sample survey for understanding facility of health service availability and readiness to offer care, understand quality of care including adherence to standards and patient outcomes, and critically assess quality of care. The EMFK, in 2018, undertook the project47 inventory mapping that aimed to assess the capacity, readiness and ability of public hospitals (both government and mission hospitals) to provide emergency surgical services in Kenya. Information on equipment, human resource and adherence to surgical guidelines were collected in county-level surveys conducted between 2018 and 2019 [36]. Those not publicly available have been provided through personal communication.

### Merging the data sources

Hospitals offering VLBW services were defined as those with an infant incubator, oxygen services, X-Ray, IV fluids available, blood transfusion, having at least two medical officers (with sensitivity analysis using hospitals with at least a paediatrician) and kangaroo mother care [30, 37]. In addition, these should have the capacity to offer caesarean sections defined as those with an operating theatre, in addition to blood transfusion which is critical in providing comprehensive emergency obstetric care. Caesarean sections are particularly important for managing complications such as preterm or obstructed labour [38, 39].

Availability of each of these services was therefore extracted from all the sources and a single database of facilities including the services they provide was created using a combination of the data sources. In some cases, information on service availability was conflicting, and a process of reconciling the differences was required. SARAM was chosen as the baseline because it is the most comprehensive dataset. If a facility had the service available in the SARAM, it was adopted. Otherwise, its availability status was cross-checked with information from the EMKF and HHFA datasets. If the two more current databases showed discordance with the SARAM, those from the EMKF and or the HHFA were adopted. Facilities with no information on whether services were available or not were labelled as “don’t know”. This list was then merged with the hospital list obtained from DHIS 2 to extract facilities with data on blood transfusions administered and number of operations (as an indicator of an operating theatre available). All public, faith-based, non-government and private facilities were included in the data matching exercise. Spatial coordinates for each facility were obtained from existing master health facility geo-coding work [40–42].

### Mapping livebirths needing VLBW

Defining accessibility to health facilities in a cost distance framework requires an understanding of population distribution at fine spatial resolution. To model total population distribution, input data included the total population counts from 54,000 enumeration areas (EA) used during the national census and are often equivalent to villages. Populations were projected to 2019 using the 2009–2019 intercensal growth rates at county levels [43]. A random forest algorithm was then used to redistribute the population counts within each EA according to different land covers, elevation or night-time light intensities at 100m spatial resolution [44]. In the absence of the most recent crude birth rate data, the modelled population was then used to derive the number of livebirths using crude birth rates obtained from the 2009 census at each of the 47 counties [45].

This gridded raster surface of births in 2019 was then used with additional data on the prevalence of VLBW to estimate the fraction of births requiring emergency hospital services (S1 Text). In brief, data and literature on VLBW prevalence were obtained from published literature, the Kenya 2014 demographic and health survey and hospital surveillance data from 15 hospitals. The number of VLBW births were divided by the total number of births in the corresponding periods to obtain the prevalence of VLBW.

### Geographic accessibility

The accessibility model corrected for various landscape factors that act as either enablers or barriers towards travelling to health facilities including land use, elevation, road types, travel speeds, weather variation, water bodies and protected areas. Sources of these datasets including their spatial resolutions and other specifications are summarised in Table A in S2 Text. A cost friction surface was developed as raster layers at a 100 m spatial resolution matched to the population-at-risk surface. Travel speeds across different land covers and road segments were used to create a cost friction surface. Motorised speeds for highways and urban roads were obtained from the traffic act [46] and others calibrated by the Kenya roads board using roads speeds collected during the GPS mapping exercise. Walking speeds across different land use classes were obtained from previous studies in Kenya [47, 48], and are shown in detail in Table B in S2 Text.

Barriers to transport (major rivers, water bodies and forests) were assigned zero speeds. The influence of slope on walking speeds was accounted for by the Tobler’s hiking equation [49]. Accessibility was computed to the geo-coded VLBW hospitals. The models were run for different months to account for the influence of seasonal weather conditions. To define inability to use road transport in areas where monthly rainfall exceeded 60mm, the speed within roads in poor conditions was assumed to be reduced by between 70 and 80% based on calibrated values from a previous study in Mozambique, in the absence of empirically measured parameters in Kenya [50]. Thus, we used monthly rainfall data to calculate accessibility metrics from January through to December. Sources of rainfall data are shown in Table A in S2 Text. Therefore, temporal (monthly) maps of access to each hospital were produced but for purposes of demonstration we show the driest and wettest months in the results. The analysis was conducted in R version 3.0 using the gdistance package [51]. The cost distance model assumed a composite model of both non-motorised and motorised transport where it was assumed patients first walk to nearest roads where motorised transport was subsequently obtained. To define accessibility, the two-hour threshold was used and population within these catchments summarised at county levels. While medical urgency varies depending on medical condition, optimal geographic access to comprehensive emergency obstetric and neonatal care is considered to be within 2 hours [52, 53] and such a threshold has been used to define geographic access to inpatient newborn care in different settings [47, 48].

### Relationship between geographic access and neonatal mortality

NMR data was obtained from the EQUIST modelled outputs [54]. Briefly, the framework used county-level household survey data on modelled interventions affecting variation in neonatal mortality, to downscale national estimates of NMR to county levels using the Lives Saved Tool (LiST) tool. Interventions used in the disaggregation were water and sanitation, bed net use, access to family planning, immunization coverage, skilled birth attendance and antenatal care attendance [54].

The relationship between access to VLBW hospitals and NMR was assessed using disaggregated data at county levels. The primary variable was livebirths with VLBW living within 2 hours of a VLBW hospital, while the outcome variable was NMR per 1,000 livebirths in 2016 [55]. No time-matched, sub-national estimates of NMR are available and here we assume NMR in 2016 was comparable to 2019. Potential confounders explored included county level estimates of wealth, maternal education, adolescent fertility, urbanization and health workforce (sources and variables described in Table A in S3 Text).

### Statistical analysis

The first step involved assessment of bivariable (crude) relationships between access, confounders and NMR using a generalised linear model (glm). Potential non-linear relationships are shown in Fig A in S3 Text, prompting the use of a glm. Relationships with p>0.20 in the bivariable models were excluded from the multivariable model. In the second step, a multivariable linear regression model that defines the relationship between access and NMR while adjusting for confounders was fit using the remaining confounders. The analysis was done using the R statistical software version 3.0 using glmer package. Results of significance were interpreted for geographic access only as it is the primary variable.

## Results

### Mapped VLBW hospitals

**Fig 1:**
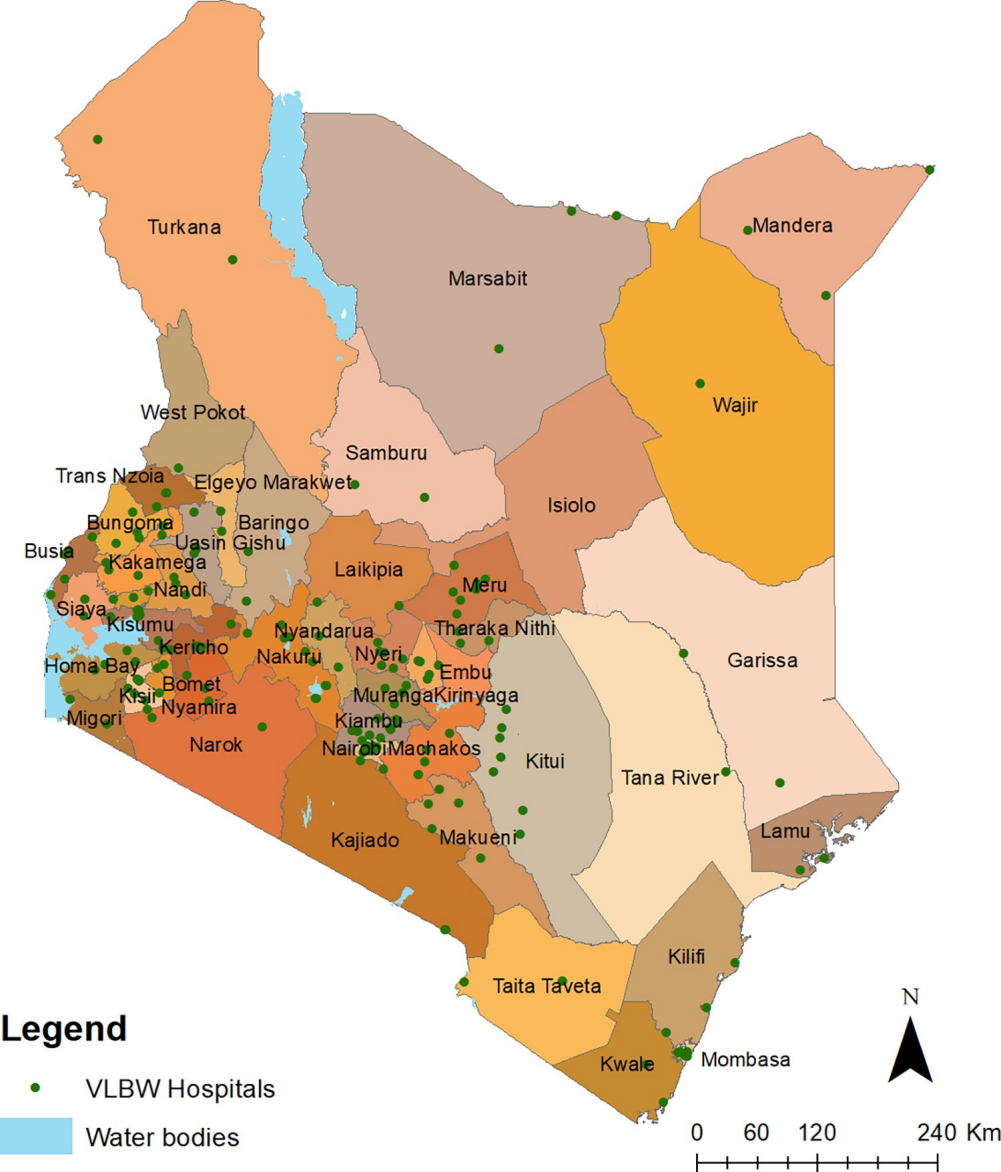
Distribution of 228 VLBW hospitals shown as green dots. The Kenya county shapefiles was obtained from humanitarian data exchange website that is maintained by OCHA [32].

Among the universe of 1980 Kenya facilities that met initial criteria for further interrogation of VLBW services, 408 had an operating theatre, 351 had an infant incubator, 302 X-Ray services, 435 oxygen provision, 440 blood transfusion services and 369 had at least two medical officers. There were different combinations of service availability across the hospitals. For example, of the facilities with an operating theatre, 356/408 had blood transfusion available, while 260/408 had access to X-Ray; of the 351 facilities with an infant incubator, 297 had oxygen available during the survey, while 238 had X-ray services. Overall, there were 228 facilities with the combination of services needed for VLBW service provision in 2019. The services were at least two medical officers, infant incubators, oxygen, ability to provide caesarean deliveries, blood transfusion, space for kangaroo mother care (KMC) and feeding support using nasogastric and intravenous tubes. The distribution of these hospitals is shown in Fig 1. Majority (54%) of services are provided by the MoH, followed by the private sector (25%) and the FBOs or NGOs (21%). The specific hospital numbers by county are shown in Table A in S4 Text. Counties with few VLBW hospital numbers were Isiolo, Tana River, Embu and West Pokot having only one VLBW hospital each. Counties with higher numbers of facilities offering VLBW services were Nairobi (n = 22), Kiambu (17), Kisumu (n = 12) and Nakuru (n = 11).

### Mapped births needing VLBW services

**Fig 2:**
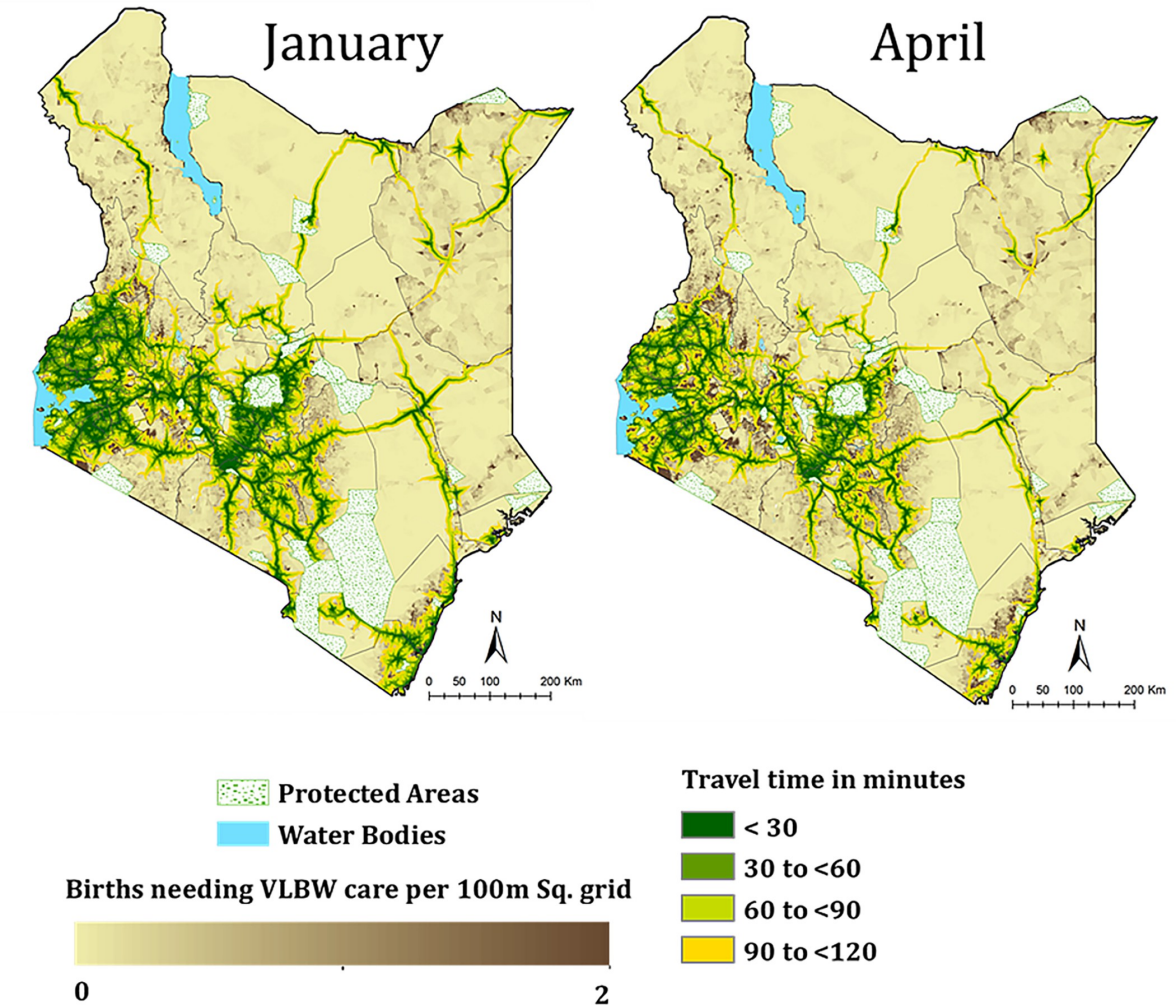
Variation in travel time to the nearest VLBW hospital showing access to VLBW hospitals in January and in April. Dark green areas are those within 30 minutes with the lighter green areas having increased travel times. Population density is also shown in areas where travel time is more than 120 minutes, while water bodies and protected areas are masked out. The Kenya county shapefiles was obtained from humanitarian data exchange website that is maintained by OCHA [32]. Maps are only shown for the wettest and driest months to highlight maximum variability.

The total projected population was 49,362,058, of these 2,123,469 were estimated as live births occurring in 2019. Using the empirical evidence of VLBW births (prevalence of 1.1%; S1 Text), it was estimated that in 2019, there were 29,729 live births born of VLBW in Kenya. The distribution of total population, and those needing VLBW services are shown in Fig A in S1 Text. A visual inspection shows that most births were occurring in areas around Nairobi, the central highlands including regions around Mount Kenya, and the western part of the country. Densely populated areas were also observed in regions *circa* 60 km from the coastline (Fig 2). Northern and North-eastern Kenya are relatively sparsely populated but there are pockets of densely populated areas particularly around major towns and refugee camps. Most births needing VLBW services were occurring in Nairobi, Nakuru, Kakamega and Bungoma counties while Lamu had the lowest expected number of VLBW births at 56.

### Geographic accessibility

**Fig 3:**
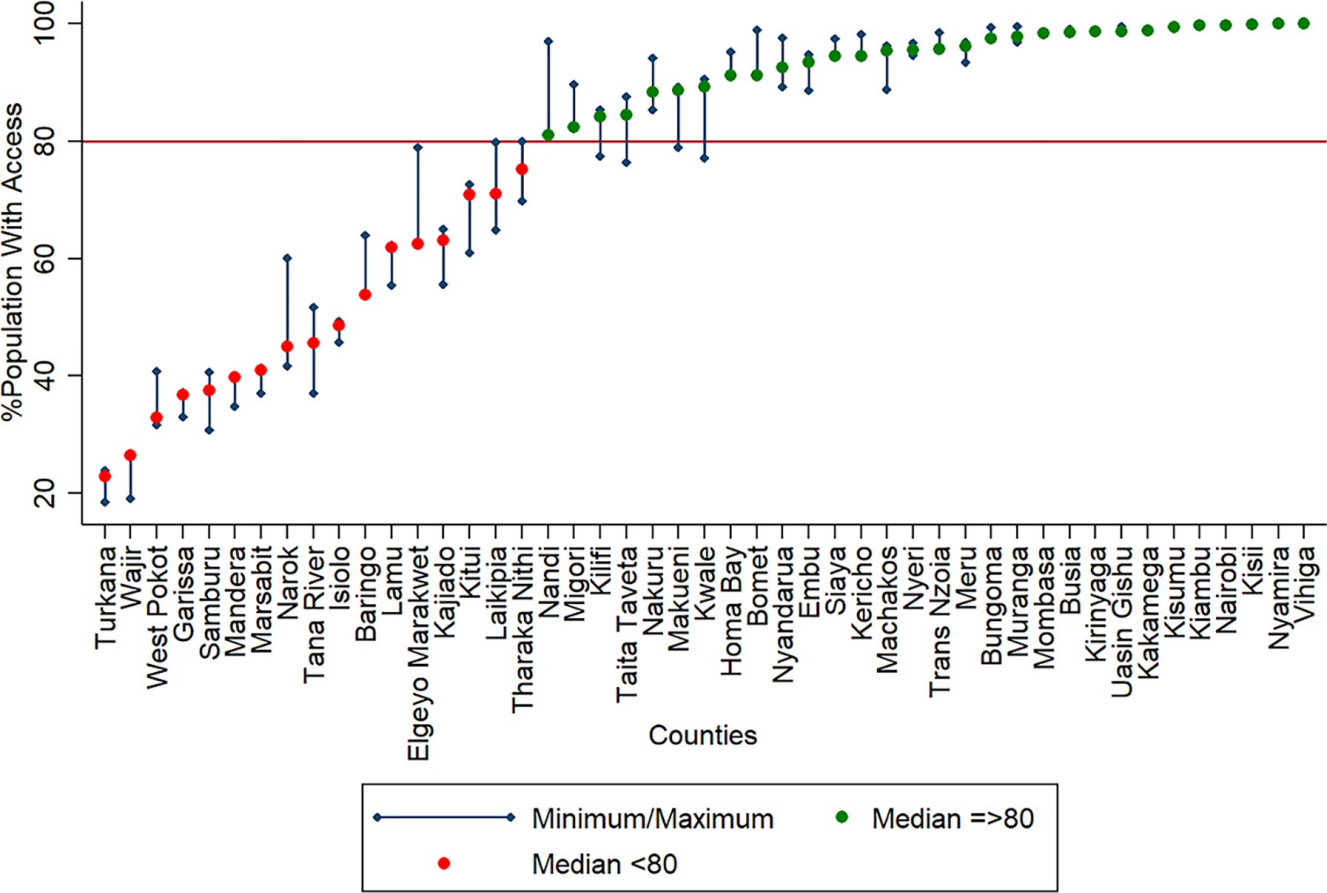
County-level variation in access to VLBW with the medians shown as dots. The error bars are the uncertainty intervals because of variation in access by seasons. The horizontal red line is the 80% cut-off. The counties are ordered by their median access quotients, with counties having less than 80% access shown in red and those with more than median 80% in green.

Nationally, 80.3% [78.0 to 83.4%] of the population in need of VLBW services were within 2 hours of the nearest VLBW hospital. Areas around northern Kenya was underserved with a significant proportion of the population in need of services outside 2 hours (Fig 2). Median access quotients (calculated from the 12 months access quotients) ranged from less than 30% in Wajir and Turkana located in the North compared to Kisumu, Kiambu, Nairobi, Kisii, Nyamira and Vihiga that had more than 80% living within 2 hours. Accessibility reduced as rainfall estimates increased, getting poorest in the rainiest month of April. Overall, 17 counties had a median access quotient less than 80%, while four counties with median access of more than 80%, did not reach this target across all the seasons (Fig 3). Table A in S4 Text shows the county level seasonal variation of access.

### Relationship between geographic access and NMR

In the initial bivariable assessment of the relationship between outcome and confounders, adolescent fertility and urbanization were dropped (Table B in S3 Text). Proximity to VLBW hospitals was associated with a reduction in NMR per 1000 livebirths, with a coefficient of -0.310 [95% CI -0.434 to -0.185] in the bivariable model. The multivariable model adjusting for wealth, health workforce density and maternal education, shows that a percent point increase in population living within 2 hours of a VLBW hospital was associated with a reduction of NMR by -0.235 [95% CI -0.463 to -0.007]. The full multivariable model coefficients are shown in Table 2.

**Table 2:** Multivariable adjusted models of the relationship between access to VLBW and NMR.

Variable	NMR_EQUIST_	
	Coefficient	p value
Intercept	42.139 [7.471 to 76.808]	0.022
Geographic access to VLBW hospitals	-0.235 [-0.463 to -0.007]	0.049
Wealth	-14.136 [-45.392 to 17.119]	0.380
Hworkforce	-0.068 [-0.641 to 0.504]	0.816
Meducation	19.399 [-13.504 to 52.302]	0.254

## Discussion

Significant differences were observed in access to hospitals with the minimum capacity to offer services to manage newborns with VLBW across Kenya and this is related to disparities in neonatal mortality across the 47 counties. The use of multiple service availability datasets allowed us to address the lack of definition of likely service availability in hospitals in previous analyses of access to comprehensive emergency care [19, 20, 56]. We determined that 228 hospitals have the necessary staffing, human resources and equipment required to manage newborns with VLBW. Accessibility results show that one in five newborns likely to need these crucial services live more than two hours from the nearest hospital, translating to approximately 6,000 VLBWs needing the emergency service being marginalised. Access varied between and within counties ranging from less than 30% in Wajir to universal access achieved in six counties.

Employing multiple criteria as minimum requirements to identify facilities capable of providing VLBW services highlights the more general issue of consistent access to full packages of interventions in hospitals in Kenya. For example, 353 facilities had an infant incubator but only 298 (84%) had oxygen while only 237 (67%) of these had chest X-ray services. Oxygen, for example, is critical in managing newborns with very low birthweight who commonly present with respiratory distress syndrome or transient tachypnoea [57]. These neonates are also prone to apnoea linked to prematurity and inadequate respiratory drive requiring them to be carefully monitored. Ensuring high quality but safe delivery of interventions such as oxygen may avert a significant proportion of neonatal deaths but needs to be part of comprehensive approaches that include other material and skilled human resources even as efforts are made to further scale up access to immediate Kangaroo Mother Care [31]. Thus, service availability censuses that only report availability of individual interventions or resource items will overestimate access when multiple interventions are required to deliver a service holistically.

We could not examine the role played specifically by improved referral or pre-referral care, ambulatory services or other multisectoral interventions such as improving road networks in the country [58]. These require more detailed and comprehensive interrogation. Nevertheless, our results suggest that a one percent increase in access to VLBW hospitals is associated with a reduction of 0.24 neonatal deaths per 1,000 livebirths, highlighting the importance of bridging physical accessibility gaps to hospital services. Counties such as Turkana, West Pokot, Wajir, Mandera and Marsabit, have less than 50% of their births occurring within 2 hours of the nearest VLBW hospitals. These counties account for only 9% of the national livebirths with most of their populations sparsely distributed, but the need for equity, leaving no one behind, demands increased investments in these counties to reduce service accessibility gaps. Improving accessibility in the marginalised counties, therefore, requires concerted efforts that focus primarily on improving services in existing facilities not meeting the minimum criteria for service provision [52, 59].

Current estimates attribute 21–32% of all neonatal deaths to be due to poor quality maternal and newborn care, so improving quality will be key to achieving the SDGs [60]. In counties with poor access including where this is linked temporally to seasonality improving road networks and referral transportation may be an important initial intervention. However, this is unlikely to have major impacts unless appropriate material and human resources are also available. In areas where geographic access is good, a focus on improving the quality of care (including referral processes) will likely be key to further reductions in neonatal mortality [61]. Providing temporal and spatial disaggregation of accessibility metrics linked to data on facility capacity offers an opportunity to identify the most useful strategies, which is key in allocating limited resources in low- and middle-income countries. In doing this it is critical to remember also the key roles of improving access to skilled birth attendance and antenatal care as important contributing factors to NMR reduction [62]. Finally, the role of reducing financial barriers towards accessing services is also important [63, 64].

This analysis had several limitations. First, in refining the definition of hospitals, secondary data was used where in some cases only availability was reported while unavailability was not always reported. Thus, it was not possible to distinguish between missing data or unavailability in all facilities and this could have an impact on the defined hospitals. Nonetheless, triangulation from different sources and data types was used to reduce the influence of this limitation. Second, hospital service availability does not equate to access to quality health care. While a facility may have the necessary capacity, these structural indicators of quality ignore the important role of processes in high-quality service provision [59]. For example, a facility may have all the necessary resources but in inadequate quantities to cope with excess workloads or be unable to deliver care effectively at night or the weekend [65, 66]. Third, some indicators such as staff availability may also be subject to variations from time to time and this may affect the selection of hospitals. For example, our minimum criteria included the presence of general medical doctors and not trained paediatricians who would normally be expected to lead delivery of care for VLBW infants and other sick newborns, a patient group that general medical doctors have very limited training to manage. If we based our analyses on hospitals with attending paediatrician, access reduced from 80% to 70% (Table B in S4 Text) [67]. There were also counties with poor coverage of access. Accessibility metrics such as speeds which defines the time taken to get to a facility may also be affected by other individual level factors such as decision on urgency. Finally, even the use of county-level information can mask significant heterogeneity in access at individual and community levels. Advancing these analyses will, however, require up to date and accurate data on place and cause of death in countries such as Kenya.

## Conclusion

This study shows that only 228 hospitals have the necessary staffing, equipment and infrastructure required to care for newborns with VLBW. At the national, level, geographic access, defined as being less than 2 hours to a facility with the basic resources needed to offer VLBW care, is just above a commonly used threshold of 80% of population threshold. There were however substantial variations in the accessibility metrics at county levels. These differences were further amplified when the condition of roads and seasonality were considered, highlighting the need for multi-sectoral approaches if accessibility gaps to inpatient neonatal services are to be reduced in the country. Consequently, a one percent increase in access to VLBW hospitals was associated with a reduction of 0.24 neonatal deaths per 1,000 livebirths. To reduce NMR to 12 deaths per 1,000 livebirths will likely require that all births are within two hours of a facility capable of providing care to VLBW infants and this will require persistent challenges of geographic access and poor quality of care to be addressed with a focus on equity.

## Supporting information

S1 Text Estimating newborn population. (DOCX)

S2 Text Covariates and parameters for modelling geographic access. (DOCX)

S3 Text Sources of confounding variables including their influence on NMR. (DOCX)

S4 Text County level quotients of accessibility. (DOCX)

## Abbreviations

EMFK: Emergency Medicine Foundation of Kenya
DHIS: District Health Information System
HHFA: Harmonised Health Facility Assessment
HDX: Humanitarian Data Exchange
KMC: Kangaroo Mother Care
MFL: Master Facility List
NMR: Neonatal Mortality Rate per 1,000 Livebirths
OCHA: Office for the Coordination of Humanitarian Affairs
SDG: Sustainable Development Goal
SSA: Sub-Saharan Africa
VLBW: Very Low Birthweight
