# Supplementary material for: Geospatial mapping of timely access to inpatient neonatal care and its relationship to neonatal mortality in Kenya
Source: PLOS Glob Public Health. 2022 Jun 30;2(6):e0000216. doi: 10.1371/journal.pgph.0000216 (PMC10021833; doi:10.1371/journal.pgph.0000216)

**Supplementary Information 1**

**Geospatial mapping of timely access to inpatient neonatal care and its relationship to neonatal mortality in Kenya**

Paul O Ouma^1*^, Lucas Malla^2^, Benjamin W Wachira^3^, Hellen Kiarie^4^, Jeremiah Mumo^4^, Mike English^2,5^, Robert W Snow^1,5^, Emelda A Okiro^1,5^

1. Population Health Unit, Kenya Medical Research Institute-Wellcome Trust Research Programme, Nairobi, Kenya
2. Health Services Unit, Kenya Medical Research Institute-Wellcome Trust Research Programme, Nairobi, Kenya
3. The Aga Khan University, Nairobi
4. Health Sector Monitoring and Evaluation Unit, Ministry of Health, Kenya
5. Centre for Tropical Medicine and Global Health, Nuffield Department of Clinical Medicine, University of Oxford, UK

**Estimating Newborns in Need of VLBW Hospital Services**

This section provides a detailed description of process used to map newborns needing VLBW services.

Three main data sources were used to derive the proportion of births that need VLBW services.

1. A literature review of studies reporting the prevalence of VLBW in Kenya, PubMed search terms that extract studies reporting birthweights in Kenya and these were;

“*low birth weight OR very low birth weight OR *weight OR birth* OR *weight OR VLBW or LBW OR 1500 kg OR 1.5 Kg) AND (Kenya) AND (newborns)*”

Several indicators were used to judge the quality of the studies and to estimate the weights used in calculation of the averages [Borges Migliavaca et al., 2020]. First, was whether the study reported that only registry data were used to obtain weight measurements. The second was if both VLBW numbers and total births in the facility was reported. Lastly, was whether it was recorded that weight measurements were taken within the first one hour of birth as after this period, newborns experience significant weight gains [Karim et al., 2018]. Thus, having met all three criteria meant a weight of 1, those with two weighted 0.67, the ones meeting only one weighted 0.33 and the calculation of mean VLBW prevalence accounted for these weights. The results are shown in Table 1.

1. Two Health and Demographic Surveillance Systems (HDSS)

The two HDSS datasets used were; Kilifi Health and Demographic Surveillance System (KHDSS) [Scott et al., 2012], and the Nairobi Urban Health and Demographic Surveillance System (NUHDSS) [Beguy et al., 2015]. The HDSS is a platform for investigating the health of individuals within a specific area, often serving as sources of data for studies evaluating the impacts of interventions. The number of children born and their characteristics including weight are normally enumerated at the household level. The Kilifi HDSS data was obtained through the KEMRI-Wellcome Trust internal data request process, while summaries from the NUHDSS was obtained from the published data from the NUHDSS [Mutua et al., 2015].

1. Extracting same numbers from the Clinical Information Network (CIN)

The CIN is a collaboration between public hospitals, policymakers and researchers and aims to improve availability of information on the quality of inpatient paediatric care [Ayieko et al., 2011]. In April 2018, the CIN started collecting data on inpatient neonatal care and this included preterm births admitted to the newborn wards. Information on birthweights was therefore reported and those born with VLBW were extracted for 16 hospitals with data. Summaries of data sources are shown in Appendix 3. VLBW prevalence was therefore calculated by multiplying the proportion of VLBW births with the total number of births surface.

A total of 111,351 births were extracted from all the studies of which 2,342 were VLBW. From each study, prevalence of VLBW varied from 0.80% in the DHS data to 3.16% in CIN hospital 9. All sources were obtained from registry data/birth cards. None of the sources from CIN reported total facility births and these had to be obtained from DHIS2. In four sources, it was not possible to determine whether birthweights were obtained within one hour of birth as shown in SI 1 Table 1. The calculated prevalence of 0.011 was therefore applied to the births surface to determine those in need of VLBW services. The final population surface is shown in SI 1 Figure 1,

.

Table A S1 Characteristics of studies and data used to estimate the prevalence of very low birth weight in Kenya. The table shows information on the number of children with VLBW, the total number of livebirths and the year the data represents.

| **Source** | **Year** | **Setting** | **VLBW %** | **Registry data/Card?** | **Total birth weights reported** | **Reported weight collected within 1 hour** | **Total births** | **Number VLBW (<1500g)** |
| --- | --- | --- | --- | --- | --- | --- | --- | --- |
| KHDSS | 2018 | KDSS | 1.53% | Y | Y | Y | 2,744 | 42 |
| [Mutua et al., 2015] | 2015 | NUDSS | 1.10% | Y | Y | Y | 4,389 | 48 |
| DHS | 2013 | Village | 0.80% | Y | Y | N | 4,184 | 334 |
| Were et al | 2009 | 3 hospitals | 1.90% | Y | N | N | 13,684 | 260 |
| Aluvaala et al | 2015 | 22 hospitals | 1.62% | Y | Y | N | 3,826 | 62 |
| Odhiambo et al | 2012 | 2 hospitals | 1.47% | Y | Y | N | 7,623 | 112 |
| CIN H1 | 2018, 1 month | 1 Hospital | 2.44% | Y | N | Y | 492 | 12 |
| CIN H2 | 2018, 9 months | 1 Hospital | 3.40% | Y | N | Y | 3,260 | 111 |
| CIN H3 | 2018, 9 months | 1 Hospital | 1.79% | Y | N | Y | 4,247 | 76 |
| CIN H4 | 2018, 1 month | 1 Hospital | 2.58% | Y | N | Y | 581 | 15 |
| CIN H5 | 2018, 9 months | 1 Hospital | 2.78% | Y | N | Y | 4,278 | 119 |
| CIN H6 | 2018, 9 months | 1 Hospital | 2.50% | Y | N | Y | 2,755 | 69 |
| CIN H7 | 2018, 1 month | 1 Hospital | 2.23% | Y | N | Y | 761 | 17 |
| CIN H8 | 2018, 9 months | 1 Hospital | 1.53% | Y | N | Y | 2,884 | 44 |
| CIN H9 | 2018, 9 months | 1 Hospital | 3.16% | Y | N | Y | 5,290 | 167 |
| CIN H10 | 2018, 9 months | 1 Hospital | 1.96% | Y | N | Y | 5,880 | 115 |
| CIN H11 | 2018, 9 months | 1 Hospital | 1.53% | Y | N | Y | 9,103 | 139 |
| CIN H12 | 2018, 9 months | 1 Hospital | 2.21% | Y | N | Y | 4,977 | 110 |
| CIN H13 | 2018, 9 months | 1 Hospital | 2.31% | Y | N | Y | 3,720 | 86 |
| CIN H14 | 2018, 12 months | 1 Hospital | 1.36% | Y | N | Y | 17,910 | 243 |
| CIN H15 | 2018, 1 month | 1 Hospital | 2.60% | Y | N | Y | 732 | 19 |
| CIN H16 | 2018, 9 months | 1 Hospital | 1.67% | Y | N | Y | 2,389 | 40 |
| KDH | 2018, 12 months | 1 Hospital | 1.81% | Y | Y | Y | 5,642 | 102 |

Fig A S1: Distribution of A) Population distribution in Kenya in 2018 showing increasing numbers from yellow to dark blue regions. The red dots represent major towns in the country B) Newborns requiring VLBW services with increasing numbers from light brown to dark brown. The boundary data were obtained from humanitarian data exchange platform (https://data.humdata.org/dataset/cod-ab-ken).


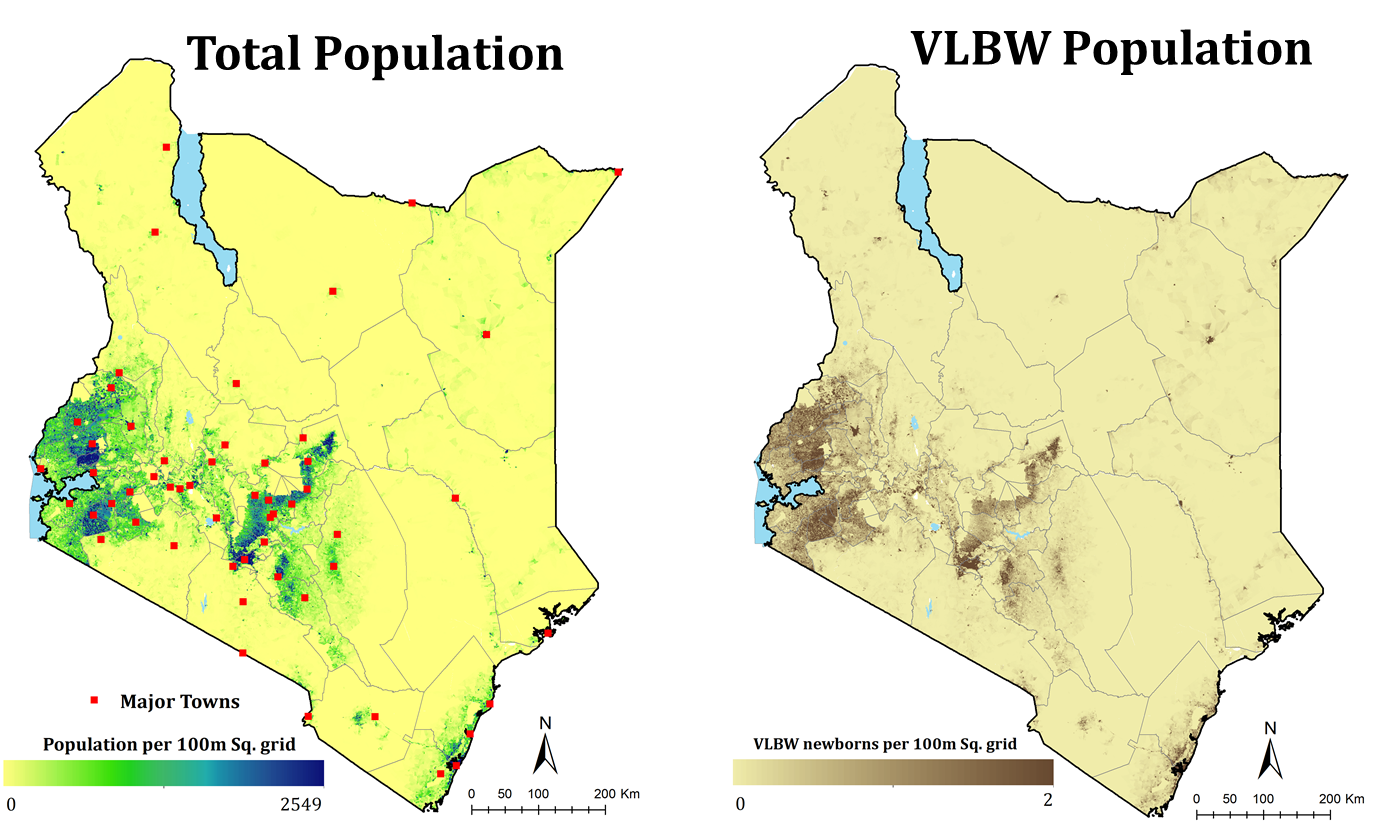

Supplement: S1 Text — (DOCX) [file pgph.0000216.s001.docx]
