# Supplementary material for: Geospatial mapping of timely access to inpatient neonatal care and its relationship to neonatal mortality in Kenya
Source: PLOS Glob Public Health. 2022 Jun 30;2(6):e0000216. doi: 10.1371/journal.pgph.0000216 (PMC10021833; doi:10.1371/journal.pgph.0000216)
Supplement: S2 Text — (DOCX) [file pgph.0000216.s002.docx]

**Supplementary Information 2**

**Geospatial mapping of timely access to inpatient neonatal care and its relationship to neonatal mortality in Kenya**

Paul O Ouma^1*^, Lucas Malla^2^, Benjamin W Wachira^3^, Hellen Kiarie^4^, Jeremiah Mumo^4^, Mike English^2,5^, Robert W Snow^1,5^, Emelda A Okiro^1,5^

1. Population Health Unit, Kenya Medical Research Institute-Wellcome Trust Research Programme, Nairobi, Kenya
2. Health Services Unit, Kenya Medical Research Institute-Wellcome Trust Research Programme, Nairobi, Kenya
3. The Aga Khan University, Nairobi
4. Health Sector Monitoring and Evaluation Unit, Ministry of Health, Kenya
5. Centre for Tropical Medicine and Global Health, Nuffield Department of Clinical Medicine, University of Oxford, UK

**Covariates used in modelling geographic access**

The accessibility model corrects for various landscape factors that act as either enablers or barriers towards travelling to health facilities. These are land use, elevation, roads, weather variation, water bodies and protected areas. Sources of these datasets including their spatial resolution and other specifications are summarised in SI 2 Table 1 and mapped in SI 2 Figure 1.

Table A S2. Covariates used in modelling spatial accessibility including their type, sources and spatial resolution.

| **Data Layer** | **Format** | **Spatial resolution** | **Purpose** | **Source** |
| --- | --- | --- | --- | --- |
| Land Use | Raster | 20m | Provide land feature classes (i.e., forestland, grassland, cropland, settlement, wetland) | sentinel 2 satellite sensor was downloaded from (https://www.rcmrd.org/) |
| Elevation | Raster | 30m | To define slope  used for adjustment of walking speed | Shuttle Radar Topography Mission downloaded from (http://gdex.cr.usgs.gov/gdex/) |
| Rainfall | Raster | 1000m | Provide rainfall estimates used to adjust speed in wet period for roads affected. | Rainfall Estimation (RFE) downloaded from (http://earlywarning.usgs.gov/fews/product/119#download). |
| Roads | Vector | NA | Provide road networks where motorized transport is enabled. With road classed defined in Appendix 4 | Ministry of Transport, Infrastructure, Housing & Urban Development maps digitised |
| Protected Areas | Vector | NA | Provide a layer of barrier to travel | KWS and KFS obtained from (http://biodiversityatlaskenya.org/bio-geoportal/) |
| Water bodies (Rivers and lakes) | Vector | NA | Provide layers of barriers to travel | Global Lakes and Wetlands Database from (http://www.worldwildlife.org/pages/global-lakes-and-wetlandsdatabase) |

Fig A S2. A) Kenya land uses as shown in different colours. B) Digital elevation model showing increasing elevation from light brown to dark brown. C) Kenya road network showing the different classes. Detailed description of the road classes is found in Appendix 4. D) Shows the barriers to travel i.e, protected areas, lakes and river. E) Shows rainfall estimates in the driest month of January while F) Shows rainfall distribution in the wettest month of April. from humanitarian data exchange platform (https://data.humdata.org/dataset/cod-ab-ken).


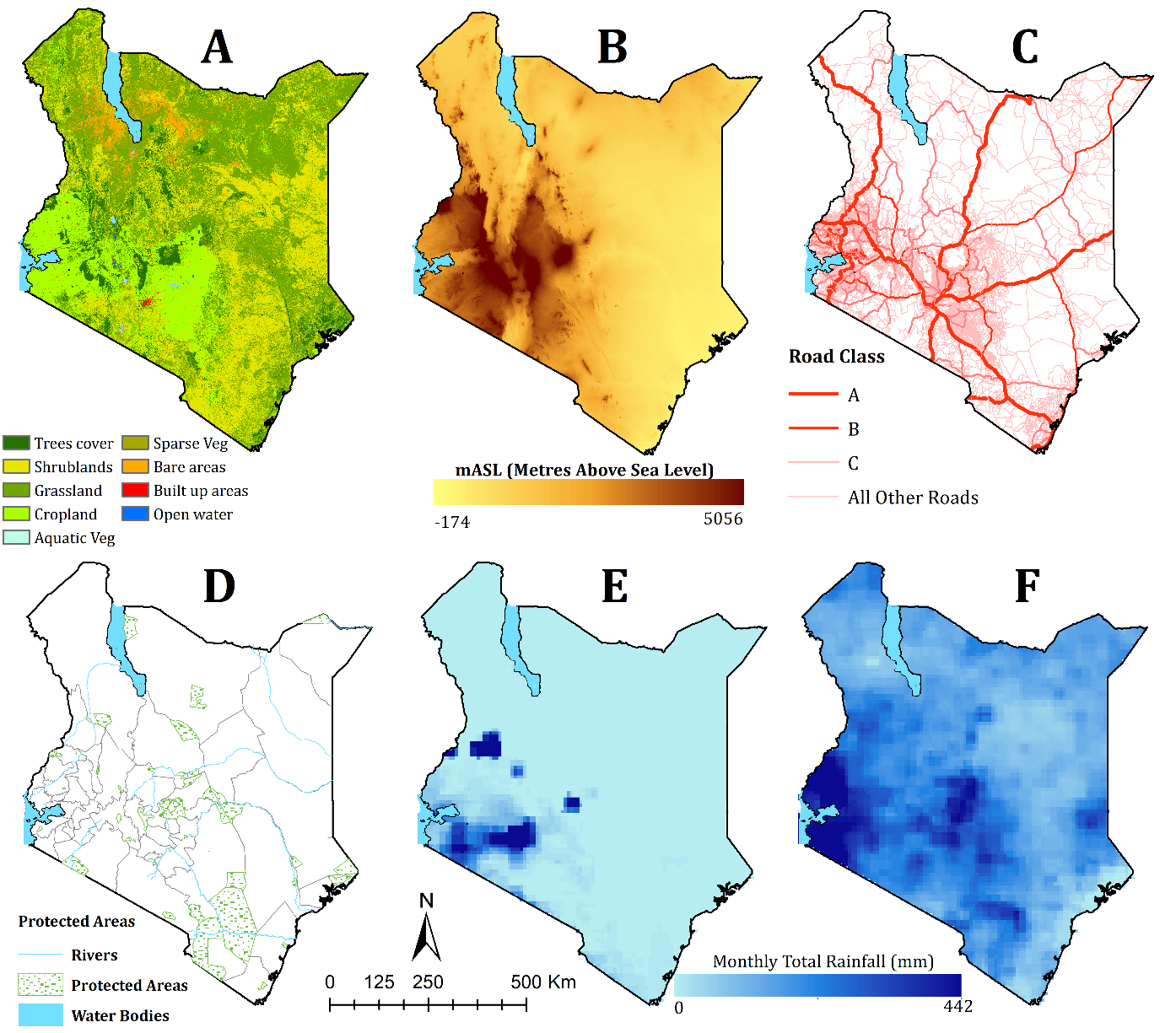


**Footnote:** In deriving the cost friction surface, all the vector layers were converted to rasters, and their spatial resolution matched to the land cover grid that was resampled to 100m spatial resolution. For the roads classified as A, B or C, motorized vehicular transport was assumed. Along tertiary classified roads, it was assumed that motorcycle transport which is a significant mode of transport in Kenya was adopted.

Table B S2**:** Transport speeds used in creating the cost friction surface. For roads, only those in poor condition were deemed impassable in the wet period.

| **Data** | **Classification** | **Speed (Km/hr.) -Dry** | **Speed (Km/hr.) - Wet** | **Model (Dry)** |
| --- | --- | --- | --- | --- |
| Land Use | Cultivated Land | 5 | 4 | Walking |
|  | Tree cover | 5 | 4 | Walking |
|  | Grassland | 5 | 4 | Walking |
|  | Shrubland | 4 | 3 | Walking |
|  | Wetland | 2 | 2 | Walking |
|  | Urban Areas | 5 | 4 | Walking |
|  | Water Bodies | 0 | 0 | Walking |
|  | Forest | 0 | 0 | Walking |
| Roads | Class S | 110 | 88 | Driving |
|  | Class A | 80 | 64 | Driving |
|  | Class B | 60 | 48 | Driving |
|  | Class H | 50 | 40 | Driving |
|  | Class J | 50 | 40 | Driving |
|  | Class C | 50 | 40 | Driving |
|  | Class D | 40 | 32 | Driving |
|  | Class E | 30 | 24 | Driving |
|  | Class F | 30 | 24 | Driving |
|  | Class G | 20 | 16 | Driving |
|  | Class K | 20 | 16 | Driving |
|  | Class L | 20 | 16 | Driving |
|  | Class M | 15 | 12 | Driving |
|  | Class R | 5 | 4 | Walking |
|  | Class T | 5 | 4 | Walking |
| DEM | Slope | W = 6 * exp {-3.5 * abs (S + 0.05)} |  | Walking |
