# Supplementary material for: Geospatial mapping of timely access to inpatient neonatal care and its relationship to neonatal mortality in Kenya
Source: PLOS Glob Public Health. 2022 Jun 30;2(6):e0000216. doi: 10.1371/journal.pgph.0000216 (PMC10021833; doi:10.1371/journal.pgph.0000216)
Supplement: S3 Text — (DOCX) [file pgph.0000216.s003.docx]

**Supplementary Information 3**

**Geospatial mapping of timely access to inpatient neonatal care and its relationship to neonatal mortality in Kenya**

Paul O Ouma^1*^, Lucas Malla^2^, Benjamin W Wachira^3^, Hellen Kiarie^4^, Jeremiah Mumo^4^, Mike English^2,5^, Robert W Snow^1,5^, Emelda A Okiro^1,5^

1. Population Health Unit, Kenya Medical Research Institute-Wellcome Trust Research Programme, Nairobi, Kenya
2. Health Services Unit, Kenya Medical Research Institute-Wellcome Trust Research Programme, Nairobi, Kenya
3. The Aga Khan University, Nairobi
4. Health Sector Monitoring and Evaluation Unit, Ministry of Health, Kenya
5. Centre for Tropical Medicine and Global Health, Nuffield Department of Clinical Medicine, University of Oxford, UK

Table A S3 Confounding variables used in the relationship assessment.

| **Group** | **Covariate (shortname)** | **Meaning** |
| --- | --- | --- |
| **Socio economic well being** | Wealth | The proportion of households classified as poor or poorer by wealth index |
| **Maternal Knowledge and autonomy** | Meducation | The proportion of mothers (15-49 years) who had less than primary education |
| **Fertility** | Adolescent_Fertility | The proportion of adolescent women age 15-19 are already mothers or pregnant with their first child |
| **Urbanization** | Urbanization | Proportion of population living in urban areas |
| **Access to healthcare** | HWorkforce | Health workforce (nurses, clinical officers, and doctors) density per 10,000 population |
|  | Geographic access to VLBW hospitals | Proportion within 2 hours of the nearest hospital |

All the variables were estimated at county levels using a smoothed small area estimation (SAE) model as described in [Macharia et al., 2019]. The data was obtained from the Kenya Demographic and Health Survey (2014). In brief, the SAE model was used to provide estimates by allowing counties with poorly sampled data to borrow from those with more information. This was done using a Bayesian gaussian process regression model that accounted for the large sampling variance and heterogeneity while exploiting spatial relatedness. The model for each variable was run using an MCMC algorithm in the R (Version 3.4.1) using 10,000 posterior samples of smoothed estimates.

The initial assessment of bivariable relationships between geographic access to VLBW hospitals, each confounder and NMR are shown in SI3 Figure 1. There was potential nonlinear relationships between the outcome and adolescent fertility and urbanization and this prompted the use of generalised linear models. The unadjusted relationship parameters are shown in SI3 Table 2.

Fig A S3 Unadjusted relationships between each variable and all the outcome. Full descriptions of the abbreviations are given in Table 1 above.


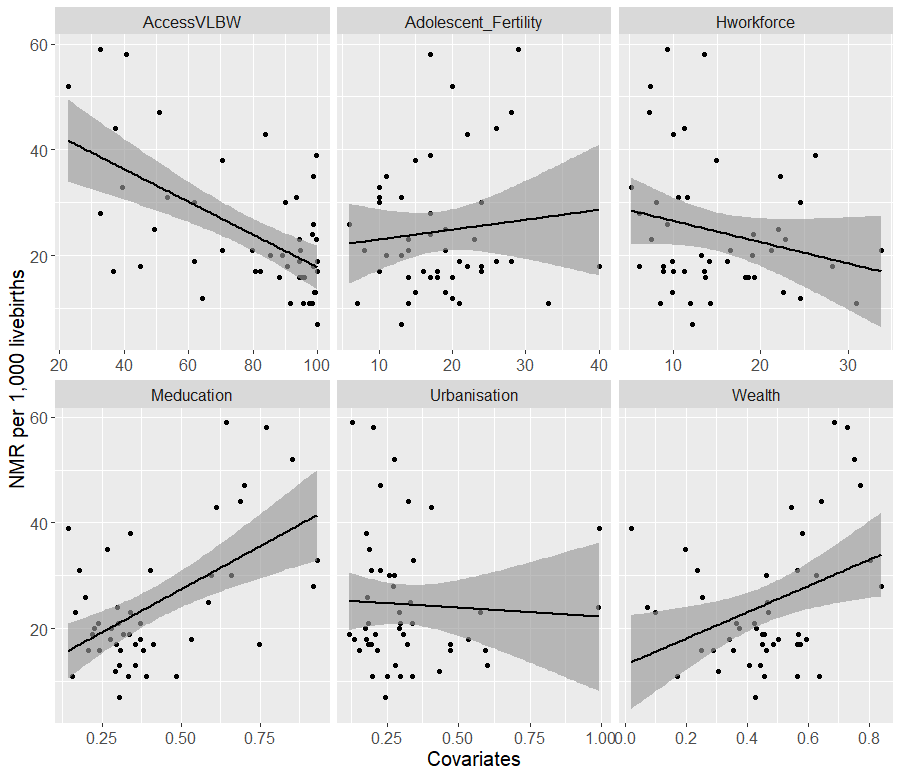


Table B S3 Unadjusted model parameters showing their respective coefficients, upper and lower bounds in brackets and p values using 95% CI. Variables which were not significant are shown with p values in bold.

|  | **NMR EQUIST** | |
| --- | --- | --- |
| **Variable** | **Coefficient** | **p Val** |
| Geographic Access | -0.330  [-0.434 to -0.185] | **<0.0001** |
| HWorkforce | -0.401  [-0.910 to 0.107] | **0.129** |
| Adolescent Fertility | 0.186  [-0.338 to 0.711] | 0.4890 |
| Wealth | 24.813  [6.6771 to 42.948] | **0.0102** |
| Meducation | 32.551  [17.769 to 47.331] | **<0.0001** |
| Urbanisation | 12.682  [-0.529 to 27.871] | 0.296 |
