# Supplementary material for: Geospatial mapping of timely access to inpatient neonatal care and its relationship to neonatal mortality in Kenya
Source: PLOS Glob Public Health. 2022 Jun 30;2(6):e0000216. doi: 10.1371/journal.pgph.0000216 (PMC10021833; doi:10.1371/journal.pgph.0000216)
Supplement: S4 Text — (DOCX) [file pgph.0000216.s004.docx]

**Supplementary Information 4**

**Geospatial mapping of timely access to inpatient neonatal care and its relationship to neonatal mortality in Kenya**

Paul O Ouma^1*^, Lucas Malla^2^, Benjamin W Wachira^3^, Hellen Kiarie^4^, Jeremiah Mumo^4^, Mike English^2,5^, Robert W Snow^1,5^, Emelda A Okiro^1,5^

1. Population Health Unit, Kenya Medical Research Institute-Wellcome Trust Research Programme, Nairobi, Kenya
2. Health Services Unit, Kenya Medical Research Institute-Wellcome Trust Research Programme, Nairobi, Kenya
3. The Aga Khan University, Nairobi
4. Health Sector Monitoring and Evaluation Unit, Ministry of Health, Kenya
5. Centre for Tropical Medicine and Global Health, Nuffield Department of Clinical Medicine, University of Oxford, UK

**Hospital numbers by counties**

The Table shows variation in hospital numbers, population and accessibility metrics by county.

Table A S4: Numbers of VLBW hospitals individually. Total number of hospitals are also shown. Percentages are calculated for the total number of hospitals.

| **County** | **VLBW Hospitals** | **Total Population** | **Livebirths** | **Livebirths needing VLBW** | **Access to all VLBW** |
| --- | --- | --- | --- | --- | --- |
| Baringo | 2 | 727,417 | 33,388 | 467 | 56.75 [53.53 to 63.64] |
| Bomet | 2 | 1,133,727 | 52,378 | 733 | 91.91 [90.77 to 98.91] |
| Bungoma | 5 | 1,998,938 | 93,550 | 1,310 | 97.76 [97.51 to 99.45] |
| Busia | 5 | 985,867 | 46,533 | 651 | 98.59 [98.53 to 99.11] |
| Elgeyo Marakwet | 2 | 476,095 | 20,853 | 292 | 66.50 [61.83 to 78.54] |
| Embu | 1 | 620,846 | 20,364 | 285 | 91.86 [88.40 to 94.76] |
| Garissa | 3 | 821,048 | 42,448 | 594 | 35.87 [32.95 to 37.18] |
| Homa Bay | 5 | 1,167,635 | 62,702 | 878 | 92.24 [91.46 to 95.56] |
| Isiolo | 1 | 200,664 | 8,448 | 118 | 48.05 [45.78 to 49.46] |
| Kajiado | 4 | 787,623 | 49,778 | 697 | 60.72 [55.24 to 64.80] |
| Kakamega | 6 | 2,293,132 | 102,732 | 1,438 | 99.02 [98.99 to 99.21] |
| Kericho | 9 | 614,335 | 33,358 | 467 | 94.75 [94.37 to 98.14] |
| Kiambu | 17 | 1,838,358 | 71,144 | 996 | 99.69 [99.53 to 99.83] |
| Kilifi | 5 | 1,462,478 | 68,736 | 962 | 81.81 [77.31 to 85.21] |
| Kirinyaga | 4 | 600,675 | 16,098 | 225 | 98.66 [98.64 to 98.68] |
| Kisii | 8 | 1,422,800 | 63,172 | 884 | 99.95 [99.94 to 100] |
| Kisumu | 12 | 1,212,277 | 54,674 | 765 | 99.51 [99.48 to 99.78] |
| Kitui | 7 | 1,243,189 | 53,208 | 745 | 67.84 [60.70 to 72.25] |
| Kwale | 2 | 808,950 | 38,425 | 538 | 84.54 [76.67 to 90.32] |
| Laikipia | 3 | 571,026 | 20,728 | 290 | 71.52 [64.46 to 79.47] |
| Lamu | 2 | 121,069 | 3,995 | 56 | 59.85 [55.30 to 62.12] |
| Machakos | 6 | 1,418,766 | 63,986 | 896 | 93.54 [88.55 to 96.13] |
| Makueni | 5 | 1,140,307 | 41,279 | 578 | 85.32 [78.48 to 89.10] |
| Mandera | 3 | 716,735 | 39,349 | 551 | 38.04 [34.56 to 39.49] |
| Marsabit | 3 | 358,047 | 16,255 | 228 | 39.80 [36.95 to 41.31] |
| Meru | 9 | 1,660,709 | 58,623 | 821 | 95.22 [93.32 to 96.81] |
| Migori | 4 | 1,285,034 | 66,179 | 927 | 83.88 [82.06 to 89.56] |
| Mombasa | 9 | 1,268,333 | 47,055 | 659 | 98.37 [98.21 to 98.53] |
| Murang'a | 4 | 1,003,693 | 23,587 | 330 | 97.99 [96.72 to 99.63] |
| Nairobi | 22 | 4,954,635 | 178,367 | 2,497 | 99.79 [99.78 to 99.79] |
| Nakuru | 11 | 2,125,299 | 85,437 | 1,196 | 88.17 [85.09 to 94.02] |
| Nandi | 3 | 1,002,211 | 43,095 | 603 | 82.24 [80.60 to 96.84] |
| Narok | 3 | 1,093,140 | 56,078 | 785 | 45.88 [41.18 to 59.35] |
| Nyamira | 3 | 592,925 | 33,500 | 469 | 99.99 [99.99 to 99.99] |
| Nyandarua | 2 | 834,716 | 29,883 | 418 | 92.80 [88.87 to 97.57] |
| Nyeri | 8 | 744,787 | 27,408 | 384 | 95.63 [94.54 to 96.73] |
| Samburu | 2 | 237,522 | 11,354 | 159 | 35.86 [30.41 to 40.22] |
| Siaya | 4 | 923,160 | 42,742 | 598 | 94.60 [94.29 to 97.51] |
| Taita Taveta | 4 | 331,170 | 10,929 | 153 | 83.32 [77.63 to 88.48] |
| Tana River | 1 | 288,353 | 12,169 | 170 | 44.26 [36.58 to 51.35] |
| Tharaka Nithi | 2 | 458,242 | 15,168 | 212 | 74.54 [69.58 to 79.75] |
| Trans Nzoia | 3 | 1,155,734 | 50,852 | 712 | 96.15 [95.42 to 98.58] |
| Turkana | 2 | 816,941 | 41,827 | 586 | 21.76 [18.31 to 23.62] |
| Uasin Gishu | 5 | 1,223,866 | 51,892 | 726 | 98.81 [98.58 to 99.59] |
| Vihiga | 2 | 753,500 | 27,804 | 389 | 100 [100 to 100] |
| Wajir | 2 | 1,198,420 | 62,558 | 876 | 31.13 [26.63 to 32.89] |
| West Pokot | 1 | 667,664 | 29,377 | 411 | 34.44 [31.35 to 40.40] |
| **Total** | **228** | **49,362,058** | **2,123,469** | **29,729** | **80.45 [78.03 to 83.53]** |

Table B S4: Comparison between accessibility to VLBW hospitals and VLBW hospitals with a paediatrician. The adjusted R^2^ using the two metrics of access was calculated as 0.91.

| **County** | **Livebirths needing VLBW** | **% Access to VLBW** | **% Access to VLBW with Paediatrician** |
| --- | --- | --- | --- |
| Baringo | 467 | 56.75 | 35.81 |
| Bomet | 733 | 91.91 | 91.91 |
| Bungoma | 1310 | 97.76 | 92.60 |
| Busia | 651 | 98.59 | 97.49 |
| Elgeyo Marakwet | 292 | 66.5 | 42.05 |
| Embu | 285 | 91.86 | 73.70 |
| Garissa | 594 | 35.87 | 34.30 |
| Homa Bay | 878 | 92.24 | 72.91 |
| Isiolo | 118 | 48.05 | 40.84 |
| Kajiado | 697 | 60.72 | 44.33 |
| Kakamega | 1438 | 99.02 | 94.52 |
| Kericho | 467 | 94.75 | 86.77 |
| Kiambu | 996 | 99.69 | 98.06 |
| Kilifi | 962 | 81.81 | 75.93 |
| Kirinyaga | 225 | 98.66 | 97.92 |
| Kisii | 884 | 99.95 | 95.20 |
| Kisumu | 765 | 99.51 | 90.37 |
| Kitui | 745 | 67.84 | 51.29 |
| Kwale | 538 | 84.54 | 79.91 |
| Laikipia | 290 | 71.52 | 37.59 |
| Lamu | 56 | 59.85 | 0.00 |
| Machakos | 896 | 93.54 | 71.48 |
| Makueni | 578 | 85.32 | 33.90 |
| Mandera | 551 | 38.04 | 0.15 |
| Marsabit | 228 | 39.8 | 0.00 |
| Meru | 821 | 95.22 | 48.30 |
| Migori | 927 | 83.88 | 33.24 |
| Mombasa | 659 | 98.37 | 86.30 |
| Murang'a | 330 | 97.99 | 59.85 |
| Nairobi | 2497 | 99.79 | 99.79 |
| Nakuru | 1196 | 88.17 | 64.70 |
| Nandi | 603 | 82.24 | 64.83 |
| Narok | 785 | 45.88 | 33.37 |
| Nyamira | 469 | 99.99 | 99.97 |
| Nyandarua | 418 | 92.8 | 85.13 |
| Nyeri | 384 | 95.63 | 83.39 |
| Samburu | 159 | 35.86 | 0.00 |
| Siaya | 598 | 94.6 | 94.60 |
| Taita Taveta | 153 | 83.32 | 59.78 |
| Tana River | 170 | 44.26 | 34.70 |
| Tharaka Nithi | 212 | 74.54 | 60.62 |
| Trans Nzoia | 712 | 96.15 | 94.43 |
| Turkana | 586 | 21.76 | 14.79 |
| Uasin Gishu | 726 | 98.81 | 81.59 |
| Vihiga | 389 | 100 | 99.97 |
| Wajir | 876 | 31.13 | 25.63 |
| West Pokot | 411 | 34.44 | 34.44 |
| **Total** | **29,729** | **80.45%** | **69.60%** |
